# The association of patient age with postoperative morbidity and mortality following resection of intracranial tumors

**DOI:** 10.1016/j.bas.2021.100304

**Published:** 2021-10-21

**Authors:** Yang Yang, Anna M. Zeitlberger, Marian C. Neidert, Victor E. Staartjes, Morgan Broggi, Costanza Maria Zattra, Flavio Vasella, Julia Velz, Jiri Bartek, Alexander Fletcher-Sandersjöö, Petter Förander, Darius Kalasauskas, Mirjam Renovanz, Florian Ringel, Konstantin R. Brawanski, Johannes Kerschbaumer, Christian F. Freyschlag, Asgeir S. Jakola, Kristin Sjåvik, Ole Solheim, Bawarjan Schatlo, Alexandra Sachkova, Hans Christoph Bock, Abdelhalim Hussein, Veit Rohde, Marike L.D. Broekman, Claudine O. Nogarede, Cynthia M.C. Lemmens, Julius M. Kernbach, Georg Neuloh, Niklaus Krayenbühl, Paolo Ferroli, Luca Regli, Oliver Bozinov, Martin N. Stienen

**Affiliations:** a Department of Neurosurgery, Cantonal Hospital of St. Gallen, St. Gallen, Switzerland; b Department of Neurosurgery, University Hospital Zurich and Clinical Neuroscience Center, University of Zurich, Zurich, Switzerland; c Department of Neurosurgery, Fondazione IRCCS Istituto Neurologico Carlo Besta, Milan, Italy; d Department of Neurology, Public Health and Disability Unit, Fondazione IRCCS Istituto Neurologico Carlo Besta, Milan, Italy; e Department of Neurosurgery, Karolinska University Hospital, Stockholm, Sweden; f Department of Clinical Neuroscience and Medicine, Karolinska Institutet, Stockholm, Sweden; g Department of Neurosurgery, Rigshospitalet, Copenhagen, Denmark; h Department of Neurosurgery, University Medical Center, Johannes Gutenberg University Mainz, Mainz, Germany; i Department of Neurosurgery, Medical University of Innsbruck, Innsbruck, Austria; j Department of Neurosurgery, Sahlgrenska University Hospital, Gothenburg, Sweden; k Institute of Neuroscience and Physiology, Sahlgrenska Academy, Gothenburg, Sweden; l Department of Neurosurgery, University Hospital of North Norway, Tromsö, Norway; m Department of Neurosurgery, St. Olavs University Hospital, Trondheim, Norway; n Department of Neurosurgery, Georg August University, University Medical Center, Göttingen, Germany; o Department of Neurosurgery, Haaglanden Medical Center, The Hague, the Netherlands; p Department of Neurosurgery, Leiden University Medical Center, Leiden, the Netherlands; q Department of Neurology, Haaglanden Medical Center, The Hague, the Netherlands; r Department of Neurosurgery, Faculty of Medicine, RWTH Aachen University, Aachen, Germany

**Keywords:** Intracranial tumor, Functional status, Outcome, Age, Risk factor, KPS

## Abstract

**Introduction:**

The postoperative functional status of patients with intracranial tumors is influenced by patient-specific factors, including age.

**Research question:**

This study aimed to elucidate the association between age and postoperative morbidity or mortality following the resection of brain tumors.

**Material and methods:**

A multicenter database was retrospectively reviewed. Functional status was assessed before and 3–6 months after tumor resection by the Karnofsky Performance Scale (KPS). Uni- and multivariable linear regression were used to estimate the association of age with postoperative change in KPS. Logistic regression models for a ≥10-point decline in KPS or mortality were built for patients ≥75 years.

**Results:**

The total sample of 4864 patients had a mean age of 56.4 ​± ​14.4 years. The mean change in pre-to postoperative KPS was −1.43. For each 1-year increase in patient age, the adjusted change in postoperative KPS was −0.11 (95% CI -0.14 - - 0.07). In multivariable analysis, patients ≥75 years had an odds ratio of 1.51 to experience postoperative functional decline (95%CI 1.21–1.88) and an odds ratio of 2.04 to die (95%CI 1.33–3.13), compared to younger patients.

**Discussion:**

Patients with intracranial tumors treated surgically showed a minor decline in their postoperative functional status. Age was associated with this decline in function, but only to a small extent.

**Conclusion:**

Patients ≥75 years were more likely to experience a clinically meaningful decline in function and about two times as likely to die within the first 6 months after surgery, compared to younger patients.

## Highlights

• A multicenter database of patients with intracranial tumors is analyzed in this study.
• Age is associated with a minor decline in the postoperative functional status & mortality.
• Patients ≥75 years are more likely to experience a clinically meaningful decline in function and to die.

## 1 Introduction

The advances in microsurgical techniques and perioperative management over the last decades allow for the safe resection of intracranial tumors in challenging locations. While the rates of acceptable postoperative functional outcome have increased owing to these technical advances, the world's population has progressively aged in parallel. As reported, 9% of the world's population is aged 65 years or older in 2019, and it is estimated that by 2050 this will increase to 16% globally and to 26.1% in Europe and Northern America (United Nations D of E and SA, 2019). The incidence of most intracranial tumors increases with age. Accordingly, neurosurgeons will face elderly patients with intracranial tumors more frequently and it is important to understand how age relates to perioperative complications and short- to mid-term outcome.

Previous studies have been inconclusive with regards to this question. While some found age to be an independent risk factor for neurological outcome, morbidity or mortality in surgically treated patients with intracranial tumors (Stark et al., 2011; Staartjes et al., 2020; Tanaka et al., 2013; Boviatsis et al., 2007), other studies failed to confirm this (Rabadan et al., 2007; Seicean et al., 2013; Stienen et al., 2018). These previous studies used heterogenous definitions of “elderly patients”, included relatively small sample sizes from single centers and focused on various different pathological entities. The goal to understand how age relates to perioperative morbidity after neuro-oncological surgery remains largely unmet. Therefore, this study aimed to elucidate the relationship of age on the short- to mid-term functional outcome after intracranial tumor surgery in a multi-center setting. We hypothesized that older age would represent an independent risk factor for perioperative functional decline after surgery.

## 2 Methods

This was a retrospective analysis of pro- and retrospectively collected data from nine tertiary neurosurgical centers in seven European countries (Supplemental Table 1). All centers pursue a “maximum safe resection” philosophy for neuro-oncological surgery. We included consecutive adult patients, who underwent microsurgical resection of intracranial tumors by craniotomy or transsphenoidal micro- or endoscopic surgery. Patients who received only biopsies were excluded. The scientific workup of data was approved by the institutional review boards (IRBs) of all contributing institutions. The study was registered at the University Hospital Zurich (http://clinicaltrials.gov, identifier NCT01628406).

### 2.1 Variables and definitions

Data of baseline characteristics included patients' age and sex. Disease-specific variables recorded were histopathological diagnosis, tumor size, prior surgery, encasement or involvement of major blood vessel or cranial nerves, as well as surgery type (craniotomy vs. transsphenoidal). The tumors were histologically classified according to previous classification system (Louis et al., 2016). The location of the lesion was classified as eloquent (motor, sensory, language, or visual areas, as well as the hypothalamus, thalamus, internal capsule, brainstem, and pineal region) versus (vs.) non-eloquent. The location was further divided into supra-vs. infratentorial space. A patient's functional status was determined by a physician using the Karnofsky Performance Scale (KPS) score at hospital admission (preoperative) and 3–6 months postoperative (Karnofsky and Burchenal, 1949). Good functional status was defined as KPS 80–100%, moderate as KPS 50–70%, and poor as KPS 0–40%. A decline of 10% or more was defined as clinically meaningful.

### 2.2 Statistical analysis

Descriptive statistics were performed on baseline demographics. Data was presented as count (percent) and mean (standard deviation) for categorical and continuous variables. The main outcome of interest (dependent variable) was the change in functional status pre-vs. postoperative, for which the preoperative KPS was subtracted from the KPS at 3–6 months postoperative (delta KPS). Age was the main independent variable of interest and both uni- and multivariable linear regression models with 95% confidence intervals (CI) were constructed to estimate the association between age with delta KPS with and without adjustment for other, potentially confounding variables.

Furthermore, we stratified the cohort into patients ≥75 years, which is a common cut-off for “late elderly”. The likelihood of these patients for pre-to postoperative functional decline of 10 points or more on the KPS or death was estimated by uni- and multivariable logistic regression, calculating (adjusted) odds ratios ((a)ORs) and 95% CIs. Sensitivity analyses were conducted, stratifying the sample into patients ​< ​or ≥65 years of age, which is the conventional definition of “early elderly” and roughly equivalent to the retirement age in most developed countries (Orimo et al., 2006; Poon et al., 2013). We did not consider life tables of the catchment areas of included hospitals, hence not analyses were run comparing the risks for mortality of tumor patients against the normal population.

Given the large sample size of the study population a p ​< ​0.006 was considered as statistically significant, to decrease the chance of type-I (false-positive) error. This critical value was based on Bonferroni correction for multiple testing with 8 degrees of freedom (0.05/8 ​= ​0.006). To detect a 5% higher likelihood for functional decline in the group of elderly patients (25 vs. 30%) with a power of 80% and alpha set a 0.006, a sample size of at least 967 patients would be required. All statistical analyses were performed using Stata (version 14.2, StataCorp, Texas, USA).

## 3 Results

### 3.1 Patient cohort

**Fig. 1:**
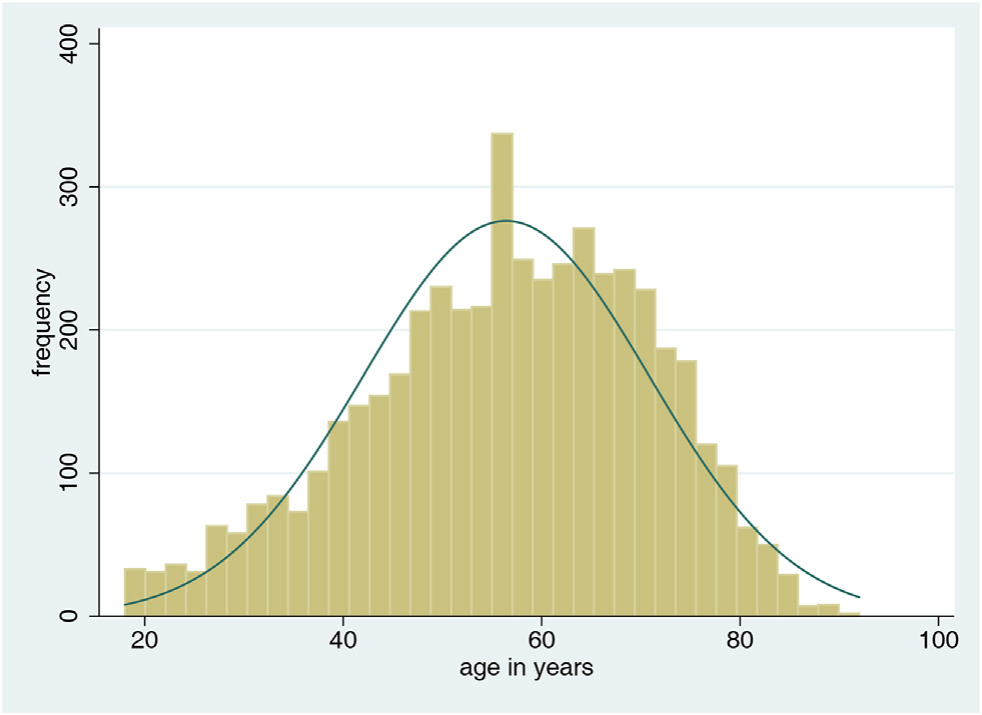
Histogram including normal curve, illustrating the age distribution (x-axis) of the total cohort (n ​= ​4864). Y-axis: Frequency.

The total sample of n ​= ​4864 patients had a mean age of 56.4 years (SD 14.4; Fig. 1), 54.5% were female. Three quarters of the patients were in good functional status at admission (KPS 80–100%). Most patients were treated for either a meningioma (40.5%), glioblastoma (21.5%) or a metastasis (12.0%). Further patient- and disease-specific characteristics are outlined in Table 1.

**Table 1:** Baseline characteristics of patients with intracranial tumors.

Variable	Value
Age (in years)	56.4 (14.4)
Sex	
Female	2653 (54.5%)
Male	2199 (45.2%)
Unknown	12 (0.3%)
Tumor diameter (in cm)	3.6 (1.7)
Histology	
Meningioma	1969 (40.5%)
Glioblastoma	1046 (21.5%)
Metastasis	583 (12.0%)
Adenoma	341 (7.0%)
Low grade glioma	168 (3.5%)
Schwannoma	155 (3.2%)
Anaplastic astrocytoma	160 (3.3%)
Craniopharyngioma	45 (0.9%)
(Epi-)Dermoid cyst	36 (0.7%)
Chordoma	24 (0.5%)
Other	337 (6.9%)
Admission KPS	
Good (80–100%)	3683 (75.7%)
Moderate (50–70%)	1090 (22.4%)
Poor (10–40%)	90 (1.9%)
Compartment	
Supratentorial	4088 (84.1%)
Infratentorial	775 (15.9%)
Eloquent location	
No	2788 (57.3%)
Yes	2075 (42.7%)
Brain vessel manipulation	
No	2786 (59.5%)
Yes	1893 (40.5%)
Cranial nerve manipulation	
No	3477 (74.3%)
Yes	1202 (25.7%)
Repeat surgery	
No	3986 (82.0%)
Yes	876 (18.0%)
Type of surgery	
Open craniotomy	4474 (92.0%)
Transsphenoidal	390 (8.0%)
**Total**	**4864 (100%)**

Values are presented as count (percent) or mean (standard deviation).

#### 3.1.1 Relationship of age with delta KPS

**Fig. 2:**
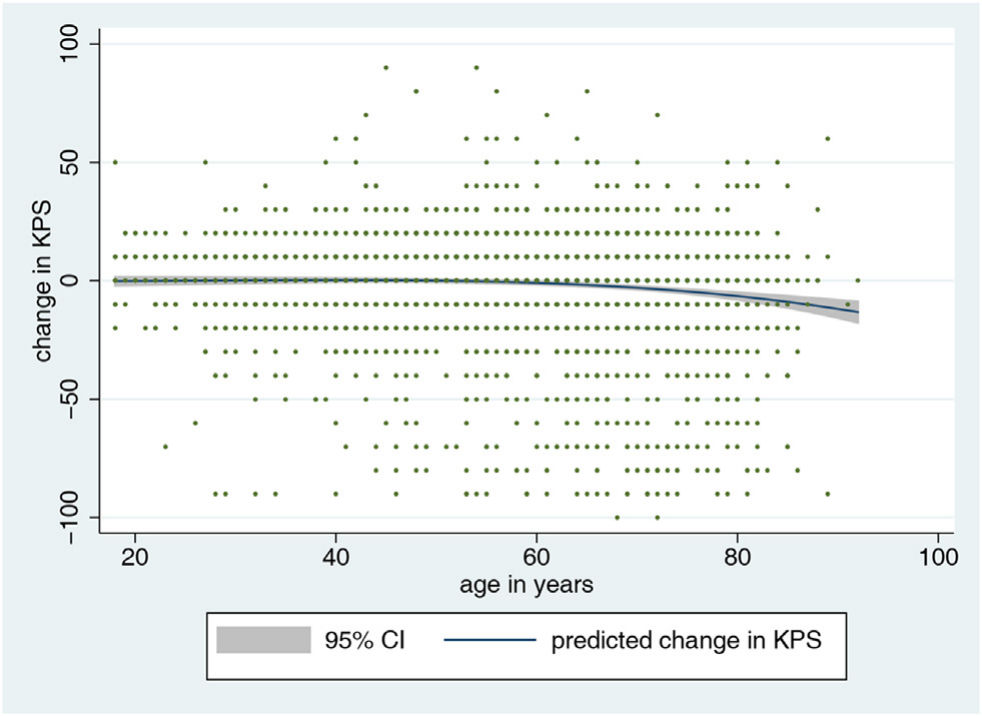
Fractional polynomial plot with 95% CI, illustrating the relationship between patient age (x-axis) and postoperative change in KPS (y-axis).

Across the whole cohort, the mean change in KPS was −1.43 (SD 19.3, range -100 – 90) between pre- and postoperative. For each 1-year increase in patient age, the decline in postoperative KPS was −0.12 points (Coeff −0.12, 95% CI -0.15 to −0.08, p ​< ​0.001). Besides, patient gender, tumor diameter and histology, admission KPS, eloquent location, brain vessel or cranial nerve manipulation, as well as type of surgery were variables found to be significantly associated with postoperative change in KPS (p ​< ​0.05). In a multivariable linear regression model, adjusted for these potential confounders, the decline in postoperative KPS was −0.11 points for each 1-year increase in patient age (Coeff −0.11, 95% CI -0.14 to −0.07, p ​< ​0.001). Fig. 2 displays in a fractional polynomial plot that the change in KPS in relation to age remains relatively stable between 20 and 60 years, but patients beyond this age show a progressive decline in postoperative function.

### 3.2 Relationship of a 75-year age cut-off with postoperative functional decline

The total sample included 479 patients ≥75 years (baseline characteristics are shown in Supplemental Table 2), of which 168 (35.1%) showed a postoperative decline in functional status (vs. 1126/4385 (25.7%) of patients <75 years, p ​< ​0.001). In univariable analysis, patients ≥75 years had an OR of 1.56 (95% CI 1.28–1.91, p ​< ​0.001) to experience postoperative decline in functional status, compared to their younger counterparts (Table 2). The effect size remained stable and statistically significant in adjusted multivariable analyses (aOR 1.51, 95% CI 1.21–1.88, p ​< ​0.001). Sensitivity analyses with an age cut-off of ≥65 years indicated the same effect but with a smaller effect size (Supplemental Table 3).

**Table 2:** Logistic regression model, estimating the likelihood of patients aged ≥75 years to experience postoperative functional decline on the KPS. The model is presented as both univariable and adjusted multivariable model.

Variable	Univariable model			Multivariable model		
	OR	95% CI	p-value	OR	95% CI	p-value
Age	1.56	1.28–1.91	<0.001	1.51	1.21–1.88	<0.001
Sex	1.34	1.18–1.52	<0.001	1.14	0.98–1.31	0.073
Tumor diameter	1.11	1.07–1.15	<0.001	1.05	1.00–1.10	0.032
Tumor histology^a^						
Glioblastoma	2.44	2.07–2.87	<0.001	2.21	1.84–2.66	<0.001
Metastasis	2.32	1.90–2.83	<0.001	1.47	1.14–1.89	0.003
Adenoma	0.25	0.16–0.39	<0.001	0.28	0.17–0.48	<0.001
Admission KPS category	0.70	0.61–0.81	<0.001	0.57	0.49–0.67	<0.001
Eloquent location	1.23	1.08–1.39	0.002	1.14	0.98–1.31	0.072
Brain vessel manipulation	0.84	0.74–0.97	0.014	0.97	0.83–1.13	0.721
Cranial nerve manipulation	0.80	0.69–0.94	0.005	1.15	0.96–1.39	0.131
Type of surgery	3.22	2.32–4.46	<0.001	1.45	0.97–2.17	0.066

a Meningioma is used as a reference (no tumor entities are excluded, but we only list the three most frequent tumor types here).

### 3.3 Relationship of a 75-year age cut-off with postoperative mortality

In patients ≥75 years, mortality rates were 37/479 (7.7%) compared to 123/4385 (2.8%) in patients <75 years (p ​< ​0.001). Patients ≥75 years had an OR of 2.9 (95% CI 1.98–4.24, p ​< ​0.001) to die at 3–6 months (Table 3) in univariable analysis, compared to their younger counterparts. The effect size was slightly attenuated in the adjusted multivariable model, but the finding remained statistically highly significant (aOR 2.04, 95% CI 1.33–3.13, p ​= ​0.001). Again, sensitivity analyses with an age cut-off of ≥65 years indicated robustness of the model (Supplemental Table 4).

**Table 3:** Logistic regression model, estimating the likelihood of patients aged ≥75 years to die at 3–6 months. The model is presented as both univariable and adjusted multivariable model.

Variable	Univariable model			Multivariable model		
	OR	95% CI	p-value	OR	95% CI	p-value
Age	2.90	1.98–4.24	<0.001	2.04	1.33–3.13	0.001
Sex	1.38	1.01–1.89	0.004	1.03	0.73–1.45	0.880
Tumor diameter	1.25	1.15–1.36	<0.001	1.13	1.02–1.26	0.025
Tumor histology^a^						
Glioblastoma	5.86	3.59–9.57	<0.001	6.49	3.77–11.1	<0.001
Metastasis	9.03	5.45–15.0	<0.001	13.7	7.78–24.1	<0.001
Adenoma	0.26	0.03–1.94	0.189	0.84	0.10–7.40	0.878
Admission KPS category	3.39	2.65–4.34	<0.001	2.93	2.23–3.86	<0.001
Eloquent location	1.19	0.87–1.63	0.275	0.83	0.58–1.17	0.289
Brain vessel manipulation	1.15	0.83–1.59	0.408	1.59	1.10–2.30	0.013
Cranial nerve manipulation	0.41	0.25–0.66	<0.001	0.89	0.51–1.56	0.690
Type of surgery	7.10	1.75–28.8	0.006	2.62	0.56–12.2	0.221

a Meningioma is used as a reference (no tumor entities are excluded, but we only list the three most frequent tumor types here).

## 4 Discussion

In this study, we analyzed the association of patient age with postoperative morbidity as measured by the KPS and mortality following microsurgical resection of intracranial tumors. Our analyses revealed that patients with intracranial tumors treated surgically showed an overall slight decline in their postoperative functional status. Age was associated with this decline in function, however only to a minor extent. Accordingly, we found that patients ≥75 years were more likely to experience a clinically meaningful decline in function as well as an increase in mortality, compared to younger patients with intracranial tumors.

### 4.1 Association of age with postoperative morbidity and mortality: clinically relevant?

Previous studies have indicated age as a predictor for postoperative morbidity, neurological status or prognosis in patients with intracranial tumors (Stark et al., 2011; Staartjes et al., 2020; Tanaka et al., 2013; Boviatsis et al., 2007). Our group recently developed and validated a clinical prediction model for functional impairment after intracranial tumor surgery (https://neurosurgery.shinyapps.io/impairment/), in which age was one of the predictive factors for functional impairment at 3–6 months postoperative (Staartjes et al., 2020). In this non-linear machine-learning-based model, we found the importance of patient age to be low to moderate. Contrarily, eloquent brain region, the surgical approach or histopathological tumor type were variables with a much higher importance, based on area-under-the-curve (AUC) analyses (compare Supplemental Table S2 in the article (Staartjes et al., 2020)).

Applying age as continuous variable in a linear regression model, we found a significant negative effect on the postoperative functional status – likely due to the large sample size and span across a wide age range. However, the effect size appeared small and clinically irrelevant. For each 1-year increase in age, the postoperative decline in functional status was 0.11 points on the KPS. Considering that the smallest detectable change in function on the KPS scale is 10 points, an age-difference of 90.9 years would be required to classify a patient into a worse KPS category. Hence, in a clinical scenario there would be a clinically meaningful higher risk of postoperative morbidity for a 109-year-old patient when compared to an 18-year-old patient undergoing resection of intracranial tumors – an unsurprising estimation.

Even though a linear model was used to illustrate the relationship, Fig. 2 underlines that the additional risk per life-year remains close to zero until the age of 60 and increments afterwards. Patient age is a given factor, which we cannot modify to lower the risk of surgery. For daily patient care, it is of higher practical relevance to understand how a certain age-category increases the relative risk for postoperative morbidity and mortality. In a second approach, we therefore explored this relationship by stratifying the total sample into patients younger or older than 75 years. We found that the risk for a 10-point postoperative decline in the KPS was elevated in the latter (Table 2) and a similar observation was made with regards to mortality (Table 3). The odds ratios (Table 2, Table 3) express moderate effect magnitudes (Chen et al., 2010). It can be appreciated from the tables that the comparative risk for mortality is more age-dependent than the risk for morbidity. The findings indicate that despite a slightly higher likelihood for adverse outcomes, there appears to be no reason to abandon surgical treatment in elderly patients in general.

### 4.2 Comparison of our results and previous literature

Our dataset comprised a considerable proportion of patients with intracranial meningiomas (40.5%), for which a relationship between age and postoperative morbidity and mortality was reported before. Poon et al. chose age ≥65 years as a cutoff and found significantly poorer functional outcomes in the elderly at 6 and 12 months after meningioma resection (Poon et al., 2013). It must be acknowledged, however, that in their analysis elderly patients had already been admitted to the hospital in a poorer neurological and functional status, and they harbored fewer WHO grade-I meningiomas. Steinberger et al. published similar results that revealed increased postoperative morbidity in patients >80 years after craniotomy for supratentorial meningioma resection (Steinberger et al., 2018). Their key findings were a significantly elevated risk of any complication, early death, and prolonged hospitalization in those patients. The effects, however, were smaller and insignificant for patients 61–70 years or 71–80 years, compared to the younger (Steinberger et al., 2018).

The second largest histopathological entity in our dataset was glioblastoma (21.5%), for which higher age has consistently been reported as a risk factor for unfavorable neurological status and outcome. This effect is hypothesized to result from an increase in comorbidities, but also from the resistance to adjuvant therapy and genetic aberrations (Tanaka et al., 2013; Laws et al., 2003; Young et al., 2015). Our current findings are in agreement with research by Senders and coworkers, who found that higher age was a risk factor for major postoperative complications leading to an extension in the length of stay, more reoperations, readmissions and mortality (Senders et al., 2018). The authors chose a slightly different approach than us, as they calculated the risks for 30-day major complication, prolonged hospitalization (>10 days) and mortality for each 10-year increase in patient age (Senders et al., 2018). In a cohort of malignant brain tumors, this shorter follow-up period may allow for the more accurate estimation of morbidity caused by the surgery itself considering that postoperative chemotherapy and radiation could be independent factors of a decrease in functional status.

As for intracranial metastases, the third largest proportion in our database (12.0%), age is one of the key variables included in prognostic indices such as the recursive partitioning analysis (RPA) or the graded prognostic assessment (GPA) (Caroli et al., 2011). In the RPA system, 65 years of age is used as cutoff to classify patients and estimate outcome, whereas the GPA system stratifies patient age throughout the sixth decade. Another large retrospective study showed that age >70 years was an independent predictor of mortality in patients undergoing radiotherapy (with or without surgery) (Lagerwaard et al., 1999). Thus, age appears to be a risk factor for outcome – regardless of whether surgery is performed. The current analysis allows for age-dependent risk estimates in a cohort of surgically treated patients with various types of intracranial tumors including brain metastases. Previous literature suggests the association of age with outcome (survival) is also dependent on the histopathological type of metastasis (e.g., lung or breast cancer, melanoma, renal cell or gastrointestinal cancer) (Sperduto et al., 2012). Our database, however, did not allow for more in-depth analyses with regards to the primary tumor.

There have been analyses on more heterogenous patient samples with multiple types of intracranial tumors before. An earlier analysis of our own group including 1951 patients with intracranial tumors focused on “preoperative dependency” as risk factor. Age was included as covariable in this model, stratified for 10-year steps, but no significantly higher likelihood of mortality or severe complications until 3 months postoperative were identified (Stienen et al., 2018). These findings are in agreement with the current analysis: also, in this multi-center data collection a 10-year increase in age did not translate into a significantly higher risk.

Depending on the study methodology, in- and exclusion criteria, age cutoffs/stratifications and definitions of outcome, several other previous articles did not constitute higher age as a risk factor for postoperative morbidity or mortality in patients with brain tumors (Rabadan et al., 2007; Seicean et al., 2013; Stienen et al., 2018). Rabadan et al., for example, conducted a retrospective analysis of 236 craniotomies for the resection of intracranial metastases and malignant gliomas (Rabadan et al., 2007). The authors observed insignificantly higher rates of both intraoperative complications (21.3 vs. 13.0%) and 30-day mortality (4.0 vs. 1.9%) in patients >60 years (Rabadan et al., 2007). Another report from India that included many large pediatric tumors, however, found age <18 years to be a risk factor for both morbidity (OR 2.06, 95% CI 1.05–4.01, p ​= ​0.035) and mortality (OR 4.67, 95% CI 1.00–21.6, p ​= ​0.049) (Moiyadi and Shetty, 2012). Altogether, many articles in the literature confirm a relatively minor contribution of the age variable itself to postoperative complications, morbidity, and mortality.

### 4.3 Effects of other factors on postoperative neurological status

Included variables with a larger impact on the outcome than age were the admission KPS, sex, eloquent lesion location, and type of surgery. The findings are in agreement with previous research where functional dependency (defined by the KPS) was a strong and independent predictor of postoperative complications and outcome, including mortality (Stienen et al., 2018; Senders et al., 2018). Maldaner et al. found that the KPS at hospital admission was associated with postoperative functional outcome in octogenarians (Maldaner et al., 2018). In another relative large cohort study of 1094 patients, Cinotti et al. identified vigilance (on the Glasgow Coma Scale (GCS)) as a powerful predictor for neurological complications leading to ICU management in patients undergoing brain tumor surgery (Cinotti et al., 2018). Sex differences in the incidence, therapeutic response and outcome of brain tumors are well described (Sun et al., 2015). In most studies, including the current one, female sex was found to be a protective factor for postoperative neurological outcome, but the (epi)genetic reasons behind this are not fully explored (Sun et al., 2012, 2015; Weil et al., 1998; Kelly et al., 2020). The lesion characteristics, including size, location and degree of malignancy naturally affect surgical decision-making, operative time, and the chosen surgical approach, which translates into the functional outcome after treatment. Our analyses indicate that eloquent tumor location and type of surgery were strong predictors for postoperative functional outcome, which is in agreement with the pertinent literature (Staartjes et al., 2020; Cinotti et al., 2018; Ferroli et al., 2015).

### 4.4 Strengths and limitations

This is a retrospective analysis of a reasonably large and comprehensive, mostly prospective database, allowing for robust analysis of demographics and some basic patient- and disease-specific factors. The multinational and multicenter nature of the data minimizes selection bias and improves generalizability of the current findings to other centers and settings. The large sample size allows for statistical controlling of multiple potential confounders, and the calculation of effect sizes allows for interpretation of clinical relevance.

The limitations of this study are intrinsic to the underlying database. Some salient factors of specific tumor characteristics, for example detailed anatomical tumor location, molecular or genetic tumor characteristics, residual tumor load and adjuvant therapy, were not specifically coded within the database. A potentially less aggressive attitude towards the surgical treatment of elderly patients with brain tumors may have led to a higher residual tumor load in these (Awad et al., 2017). Therefore, residual tumor load could be a confounder when analyzing the association between age and KPS – a factor, which the nature of the dataset did not allow us to statistically control for. We included several distinct tumor types (incl. pituitary tumors and meningiomas), some of which show more benign postoperative courses compared to more aggressive tumor entities. Disease-specific subgroup analyses might provide more in-depth insight how age relates to morbidity in a given tumor disease. The calculation of recursive partitioning analysis (RPA) classes would have allowed for a more exact estimation of prognosis in patients with brain metastases (Gaspar et al., 2000). It should be acknowledged that elderly is inherently more likely to die compared to younger patients, as age itself is a risk factor for mortality. We did not compare the life expectancy of included brain tumor patients with age-matched normal population data; thus, we can only report the odds ratios of morbidity and mortality for the chosen age-stratification. Moreover, the burden or comorbidities naturally increases with higher age. Such factors should ideally be considered as covariates, as they have been shown to influence the main outcome of this study (Sun et al., 2015; Awad et al., 2017; Wood et al., 2011; Heiland et al., 2018). Unfortunately, the data did not allow for in-depth analyses with regards to the above-mentioned variables. Including these in the multivariable models would have allowed us to calculate the independent effect of patient age even more accurately.

## 5 Conclusions

Patients undergoing brain tumor resection experience overall a slight decline in their functional status at 3–6 months postoperative. The decline increases slightly with increasing age, however to a minor extent before the age of 60 years. We found that patients 75 years and older had an odds ratio of 1.5 and 2 to experience a clinically meaningful decline in function and mortality, respectively, compared to younger patients. Inclusion of age in the preoperative risk stratification may enable more accurate preoperative optimization and therefore mitigate risks associated with surgical treatment.
