# Supplementary material for: The association of patient age with postoperative morbidity and mortality following resection of intracranial tumors
Source: Brain Spine. 2021 Oct 21;1:100304. doi: 10.1016/j.bas.2021.100304 (PMC9560674; doi:10.1016/j.bas.2021.100304)
Supplement: Multimedia component 1 [file mmc1.docx]

| Hospital | Timespan | Data collection |
| --- | --- | --- |
| University Hospital Zurich, Switzerland | 07/2013 – 12/2017 | prospective |
| University Medical Center Göttingen, Germany | 01/2014 – 12/2017 | prospective |
| Fondazione IRCCS Istituto Neurologico Carlo Besta Milan, Italy | 01/2014 – 12/2017 | prospective |
| Haaglanden Medical Center, The Hague & Leiden University Medical Center, The Netherlands | 01/2015 – 12/2018 | prospective |
| St. Olavs University Hospital Trondheim, Norway | 01/2007 – 12/2015 | prospective, supplemented with some retrospective data |
| Karolinska University Hospital Stockholm, Sweden | 01/2007 – 12/2015 | retrospective |
| University Medical Center, Johannes Gutenberg University Mainz, Germany | 01/2007 – 12/2018 | retrospective |

**Supplemental Table 1**

Overview of the contributing tertiary neurosurgical centers, the timespan and method of data collection.

**Supplemental Table 2**

Baseline characteristics of patients < or ≥75 years with intracranial tumors. Data is presented as count (percent) or mean (standard deviation).

|  | **Patients < 75 years** | **Patients ≥75 years** |
| --- | --- | --- |
| Age (in years) | 54.0 (13.0) | 78.7 (3.3) |
| Sex  Female  Male  Unknown | 2421 (55.2%)  1954 (44.6%)  10 (0.2%) | 232 (48.4%)  245 (51.2%)  2 (0.4%) |
| Tumor diameter (in cm) | 3.6 (1.7) | 3.9 (1.6) |
| Histology  Meningioma  Glioblastoma  Metastasis  Adenoma  Low grade glioma  Schwannoma  Anaplastic astrocytoma  Craniopharyngioma  (Epi-)Dermoid cyst  Chordoma  Other | 1793 (40.9%)  905 (20.6%)  515 (11.7%)  308 (7.0%)  163 (3.7%)  146 (3.3%)  156 (3.6%)  40 (0.9%)  34 (0.8%)  22 (0.5%)  303 (6.9%) | 176 (36.7%)  141 (29.4%)  68 (14.2%)  33 (6.9%)  5 (1.0%)  9 (1.8%)  4 (0.8%)  5 (1.0%)  2 (0.4%)  2 (0.4%)  34 (7.1%) |
| Admission KPS  Good (80 – 100)  Moderate (50 – 70)  Poor (10 – 40) | 3396 (77.5%)  918 (20.9%)  71 (1.6%) | 287 (60.7%)  172 (35.9%)  19 (4.0%) |
| Compartment  Supratentorial  Infratentorial | 3672 (83.8%)  712 (16.2%) | 416 (86.9%)  63 (13.1%) |
| Eloquent location  No  Yes | 2537 (57.9%)  1847 (42.1%) | 251 (52.4%)  228 (47.6%) |
| Brain vessel manipulation  No  Yes | 2517 (59.5%)  1711 (40.5%) | 269 (59.7%)  182 (40.3%) |
| Cranial nerve manipulation  No  Yes | 3122 (73.8%)  1106 (26.2%) | 355 (78.7%)  96 (21.3%) |
| Repeat surgery  Yes  No | 822 (18.8%)  3561 (81.2%) | 54 (11.3%)  425 (88.7%) |
| Type of surgery  Open microsurgery  Transsphenoidal surgery | 4037 (92.1%)  348 (7.9%) | 437 (91.2%)  42 (8.8%) |
| **Total** | **4385 (100%)** | **479 (100%)** |

Values are presented as count (percent) or mean (standard deviation) unless otherwise indicated.

**Supplemental Table 3**

Logistic regression model, estimating the likelihood of patients aged ≥65 years to experience postoperative functional decline on the KPS. The model is presented as both univariable and adjusted multivariable model.

| Variable | Univariable model | | |  | Multivariable model | | |
| --- | --- | --- | --- | --- | --- | --- | --- |
|  | *Coef.* | *95% CI* | *p-value* |  | *Coef.* | *95% CI* | *p-value* |
| Age | 1.43 | 1.25 – 1.63 | <0.001 |  | 1.32 | 1.14 – 1.53 | <0.001 |
| Sex | 1.34 | 1.18 – 1.52 | <0.001 |  | 1.13 | 0.98 – 1.30 | 0.092 |
| Tumor diameter | 1.11 | 1.07 – 1.15 | <0.001 |  | 1.05 | 1.00 – 1.10 | 0.030 |
| Tumor histology*  Glioblastoma  Metastasis  Adenoma | 2.44  2.32  0.25 | 2.07 – 2.87  1.90 – 2.83  0.16 – 0.39 | <0.001  <0.001  <0.001 |  | 2.20  1.44  0.28 | 1.83 – 2.64  1.12 – 1.85  0.17 – 0.48 | <0.001  0.004  <0.001 |
| Admission KPS category | 0.70 | 0.61 – 0.81 | <0.001 |  | 0.57 | 0.49 – 0.67 | <0.001 |
| Eloquent location | 1.23 | 1.08 – 1.39 | 0.002 |  | 1.14 | 0.99 – 1.31 | 0.073 |
| Brain vessel manipulation | 0.84 | 0.74 – 0.97 | 0.014 |  | 0.97 | 0.83 – 1.13 | 0.677 |
| Cranial nerve manipulation | 0.80 | 0.69 – 0.94 | 0.005 |  | 1.16 | 0.97 – 1.40 | 0.109 |
| Type of surgery | 3.22 | 2.32 – 4.46 | <0.001 |  | 1.45 | 0.98 – 2.17 | 0.066 |

* Meningioma is used as a reference (no tumor entities are excluded, but we only list the three largest tumor types here).

**Supplemental Table 4**

Logistic regression model, estimating the likelihood of patients aged ≥65 years to die at 3 - 6 months. The model is presented as both univariable and adjusted multivariable model.

| Variable | Univariable model | | |  | Multivariable model | | |
| --- | --- | --- | --- | --- | --- | --- | --- |
|  | *OR* | *95% CI* | *p-value* |  | *OR* | *95% CI* | *p-value* |
| Age | 2.80 | 2.04 – 3.85 | <0.001 |  | 2.11 | 1.49 – 2.99 | <0.001 |
| Sex | 1.38 | 1.01 – 1.89 | 0.004 |  | 0.98 | 0.70 – 1.39 | 0.927 |
| Tumor diameter | 1.25 | 1.15 – 1.36 | <0.001 |  | 1.13 | 1.01 – 1.26 | 0.032 |
| Tumor histology*  Glioblastoma  Metastasis  Adenoma | 5.86  9.03  0.26 | 3.59 – 9.57  5.45 – 15.0  0.03 – 1.94 | <0.001  <0.001  0.189 |  | 6.33  13.5  0.85 | 3.68 – 10.9  7.64 – 23.7  0.10 – 7.46 | <0.001  <0.001  0.885 |
| Admission KPS category | 3.39 | 2.65 – 4.34 | <0.001 |  | 2.94 | 2.23 – 3.87 | <0.001 |
| Eloquent location | 1.19 | 0.87 – 1.63 | 0.275 |  | 0.83 | 0.59 – 1.19 | 0.316 |
| Brain vessel manipulation | 1.15 | 0.83 – 1.59 | 0.408 |  | 1.61 | 1.11 – 2.32 | 0.012 |
| Cranial nerve manipulation | 0.41 | 0.25 – 0.66 | <0.001 |  | 0.90 | 0.52 – 1.58 | 0.721 |
| Type of surgery | 7.10 | 1.75 – 28.8 | 0.006 |  | 2.55 | 0.54 – 12.0 | 0.235 |

* Meningioma is used as a reference (no tumor entities are excluded, but we only list the three largest tumor types here).
